# TAK1 signaling in fibrosis: insights from systemic sclerosis

**DOI:** 10.3389/fimmu.2026.1960713

**Published:** 2026-10-19

**Authors:** Scott Scarneo, Robert Freeze, Swati Bhattacharyya, John Varga

**Affiliations:** 1 Department of Research and Development, Eydis Bio Inc., Durham, NC, United States; 2 Michigan Scleroderma Program, Division of Rheumatology, Department of Internal Medicine, University of Michigan, Ann Arbor, MI, United States

**Keywords:** autoimmune, fibrosis, inflammation, ssc, TAK1, therapeutic, TNF

## Abstract

Systemic sclerosis (SSc) is a complex autoimmune disease characterized by fibrosis of the skin and internal organs, vascular abnormalities, and immune dysregulation. Widespread and recalcitrant fibrosis underlies the significant morbidity and mortality of SSc. TGF-β-activated kinase 1 (TAK1), a key regulator of various cellular processes including inflammation and fibrosis has emerged as a pivotal player in the pathogenesis of fibrotic diseases such as SSc. This timely review explores the biology and multifaceted roles of TAK1 in SSc, encompassing its involvement in fibroblast activation, immune cell function, and endothelial dysfunction. Mechanistically, TAK1 integrates signals from diverse cytokines and growth factors to modulate downstream effectors such as NF-κB and MAP kinases, thereby influencing inflammation, oxidative stress, and tissue remodeling in SSc. Notably, dysregulation of TAK1 signaling pathways has been implicated in the aberrant activation of fibroblasts and the persistence of profibrotic responses characteristic of SSc. Understanding the intricate network of TAK1-mediated intracellular signaling pathways offers new insights into potential therapeutic strategies targeting this kinase for the treatment of SSc and fibrosis. This review synthesizes current knowledge and highlights promising avenues for future research aimed at harnessing TAK1 as a therapeutic target in SSc management.

## Introduction

TAK1, also known as MAP3K7, is a serine/threonine kinase that plays a pivotal role in regulating multiple intracellular signaling pathways (1). In particular, TAK1 is a key upstream regulator of nuclear factor kappa B (NF-κB) and mitogen-activated protein kinase (MAPK) signaling cascades, which are critical for inflammation, immunity, and cellular stress responses. Structurally, TAK1 consists of a kinase domain located at its N-terminus and a C-terminal region that interacts with adaptor proteins. Activation is typically initiated when TAK1 forms a complex with its regulatory proteins, TAB1, TAB2, or TAB3, which are required for its recruitment to upstream activators such as receptor-interacting protein kinases or ubiquitin chains (2–6). The structural basis for the interaction between K63-linked polyubiquitin chains and the NZF domains of TAB2 and TAB3 has been determined by crystal structure analysis. After the K63-linked polyubiquitination of RIP1, the polyubiquitin chains of RIP1 bind to the C-terminal Npl4 zinc finger (NZF) domain of TAB2 and TAB3, which allows the autophosphorylation-dependent activation of TAK1 to occur after conformational changes (4). The TAK1-TAB complex is heavily and dynamically modulated by a variety of post-translational modifications, including phosphorylation, ubiquitination, lactylation, methylation, acylation, O-GlcNAcylation, and sumoylation (7–11). These modifications play important roles in regulating the activity, stability, as well as assembly of the TAK1-TAB complex, fine-tuning the inflammatory responses (12).

The kinase activation process involves phosphorylation of key residues within the activation loop of TAK1’s kinase domain, unleashing its full catalytic activity (13). This phosphorylation is often mediated by upstream stimuli such as tumor necrosis factor-alpha (TNF-α) or interleukin-1 beta (IL-1β), which activate membrane-bound receptors and recruit TAK1-containing complexes (14). Other TAK1 pathway agonist such as TGF-β, hence TAK1’s name sake, lead to activation of TAK1, although these agonists are considered less potent than TNF (15). Following upstream agonist activation, accessory proteins such as TAB1 play a crucial role in activation by promoting autophosphorylation of TAK1 at specific serine and threonine residues (6). TAK1’s kinase domain is responsible for its enzymatic activity, while its C-terminal region ensures proper protein-protein interactions and recruitment to upstream signaling complexes (16). The modular design of TAK1 allows it to integrate signals from diverse pathways, making it a central regulator in inflammatory and stress responses.

## TAK1: pivotal role in fibrotic signaling

TAK1 is a central mediator in fibrotic signaling, orchestrating pathways that regulate inflammation, extracellular matrix production, and tissue remodeling (17, 18). Fibrosis, characterized by excessive deposition of ECM components like collagen, leads to organ dysfunction and failure (19). TAK1 integrates pro-fibrotic signals from upstream activators, such as TGF-β and inflammatory cytokines, and propagates them to downstream pathways, including NF-κB, MAPKs, and Smad-independent TGF-β signaling (**Figure 1**).

**Figure 1:**
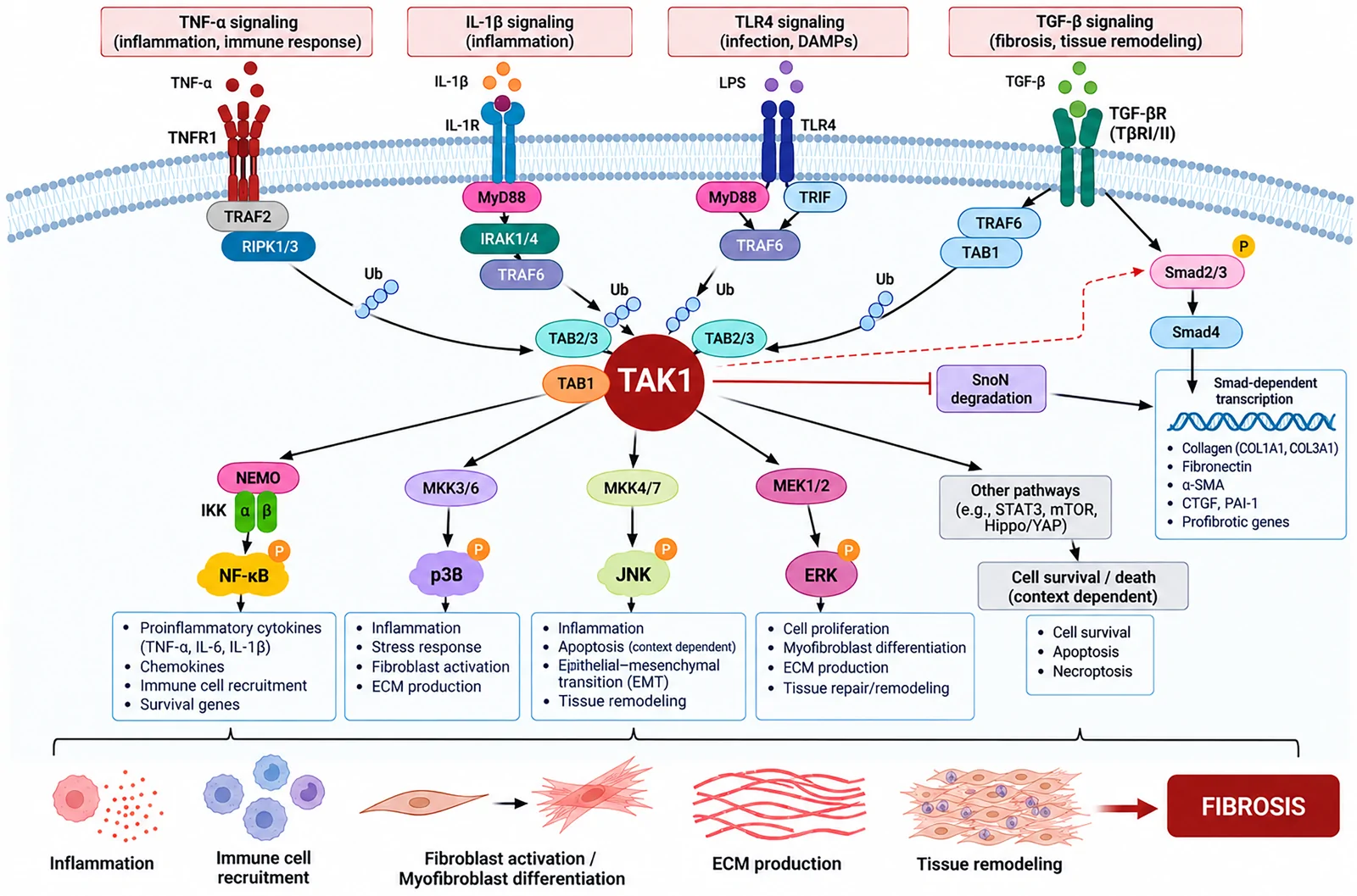
TAK1 integrates pro-inflammatory and profibrotic signaling pathways converging on fibrosis. Schematic representation of TAK1 as a central signaling hub downstream of TNFR1, IL-1R, TLR4, and TGF-β receptors. TNFR1 signaling promotes recruitment of TRAF2 and RIPK1/3, while IL-1R and TLR4 signaling recruit MyD88, IRAK1/4, TRAF6, and/or TRIF. These receptor-associated complexes promote ubiquitin-dependent recruitment of TAB proteins and activation of TAK1. TGF-β receptor signaling activates TAK1 through TRAF6/TAB-dependent mechanisms while also engaging the canonical Smad2/3–Smad4 pathway. Activated TAK1 regulates the IKK/NEMO–NF-κB axis and MAPK pathways through p38, JNK, and ERK, promoting inflammatory signaling, fibroblast activation, myofibroblast differentiation, extracellular matrix production, and tissue remodeling. TAK1 also intersects with canonical TGF-β signaling through context-dependent regulation of Smad proteins and SnoN and contributes to pathways regulating cell survival and death. Collectively, these interconnected signaling networks position TAK1 as a central molecular link between inflammation, cellular stress responses, and progressive fibrosis.

Several preclinical studies have demonstrated the important role of TAK1 signaling in fibrosis. In the context of kidney fibrosis, TAK1 has been shown to activate downstream pathways, such as the MKK3-p38 or MKK4-JNK cascades, contributing to the profibrotic effects of TGF-β1 (20). TAK1 has also been implicated in the activation of intestinal mucosal myofibroblasts, which drive gut fibrosis in Crohn’s disease (21). Outside of directly responding to TGF-β, TAK1’s role in inflammatory signaling can modulate fibrosis. For example, it has been reported that TNFα upregulated TGF-β expression in primary mouse lung fibroblasts (22). Further analysis of this pathway suggests that this is mediated through the ERK specific MAPK pathway downstream of TAK1 (22). Other research has shown that TNF signaling through TAK1 can promote fibrosis through transformation of epithelial cells to mesenchymal cells. A recent study by Li et al., shows that TNF enhances TGF-β mediated epithelia transformation leading to increased mesenchymal cells, migration and fibrosis markers (23).

Interestingly, it was previously shown that deletion of TAK1 in hepatocytes results in spontaneous development of liver inflammation and fibrosis (24). This supports that TAK1 serves as a molecular link between liver inflammation and fibrosis, however contradicts other findings where TAK1 inhibition prevented fibrosis rather than accentuated it (25). A potential explanation for this difference may be due to the scaffolding role of TAK1 which is critical in regulating protein-protein interactions that may expand beyond the main catalytic function of TAK1. Use of genetic approaches often rely on complete knockout or removal or critical segments of the TAK1 protein, leading to either conformationally null or no TAK1 expression in cells (15). The removal of TAK1 perturbs the TAK1-TAB-TRAF6 complex and may have downstream effects on signal cascades. Unlike genetic approaches, selective pharmacological TAK1 inhibition allows for TAK1 complexes to assemble and retains the scaffolding functions of TAK1. Further, pharmacological inhibition at appropriate doses may simply dial back aberrant TAK1 signaling to normal levels rather than remove all TAK1 signaling (26–29). This difference may prevent the development of compensatory pathways which may drive fibrosis seen in genetic studies. Of note, TAK1 signaling is compartmentalized into the primary cilia, the cellular signaling hub increasingly recognized at critical in modulation tissue repair and fibrosis (30).

## TAK1 and TGF-β signaling

TAK1 occupies a central position at the intersection of canonical and noncanonical TGF-β signaling, functioning as a molecular rheostat that integrates diverse inflammatory, stress, and profibrotic inputs downstream of receptor activation. Beyond serving as a parallel MAP3K signaling cascade, TAK1 dynamically modulates Smad transcriptional output through direct protein–protein interactions, post-translational modifications, and regulation of inhibitory Smads and transcriptional co-repressors (31, 32). In doing so, it coordinates the balance between transient adaptive signaling and sustained fibrogenic reprogramming. Through coupling to p38, JNK, and NF-κB pathways, TAK1 amplifies context-dependent transcriptional programs that reinforce extracellular matrix production, myofibroblast differentiation, and inflammatory crosstalk. The sections below delineate how TAK1 shapes TGF-β responses across Smad-dependent and Smad-independent axes, underscoring its role as a pivotal signaling hub in fibrosis and tissue remodeling.

### Smad-dependent pathways

TAK1 plays a critical role in regulating Smad signaling by interacting directly with the MH2 domain of Smad proteins (31). This interaction significantly alters R-Smad transactivation and affects their subcellular localization. For example, in hematopoietic myeloid cells, IL-1β transiently enhances TAK1’s association with the MH2 domain of Smad3, leading to the suppression of TGF-β-induced Smad3 signaling (33). Furthermore, TAK1 can inhibit R-Smad activation by promoting the expression of the inhibitory Smad7, further reinforcing its role as a negative regulator of R-Smad function (34).

Inhibitory Smads, notably Smad6 and Smad7, have been shown to modulate TAK1 activity in a cell-type-specific manner. For instance, in rat pheochromocytoma PC12 cells, overexpression of Smad6 or Smad7 represses BMP2-induced neurite outgrowth by inhibiting the TAK1-p38 kinase pathway (35). Conversely, in human prostate cancer PC-3U cells, Smad7 functions as a scaffold protein, facilitating TAK1-mediated activation of the MKK3-p38 signaling axis (36). Other studies of Smad7 regulation of TAK1 have shown that Smad7 enhances TGF-β’s anti-inflammatory effects by binding to TAB2 and TAB3. This interaction suppresses TNF signaling by disrupting TRAF2-TAB2/3-TAK1 complex (37). In mouse models, Smad7 overexpression reduced inflammation and NF-κB nuclear translocation by interfering with these signaling complexes (37). These findings highlight that Smad7 may regulate TAK1 inflammatory signaling through TAB2/3 modulation following TGF-β agonism.

TAK1 might also influence fibrosis through its interaction with the transcriptional co-repressor SnoN. Under basal conditions, SnoN suppresses TGF-β signaling by inhibiting Smad-dependent transcription of pro-fibrotic genes (38–41). Upon TGF-β stimulation, TAK1 phosphorylates SnoN, leading to its ubiquitination and subsequent degradation. The removal of SnoN’s inhibitory effect allows for the activation of Smad3/4 complexes, promoting the transcription of genes that drive extracellular matrix production and fibrosis (42, 43). This mechanism has been observed in various fibrotic conditions. For example, in models of diabetic nephropathy, TAK1-mediated degradation of SnoN enhances EMT and ECM accumulation, contributing to renal fibrosis (44).

Therapeutically, targeting the TAK1-SnoN axis presents a promising strategy for mitigating fibrosis. Pharmacological inhibition of TAK1 has been shown to reverse fibrogenic effects, such as those mediated by TRIM44 overexpression, by preventing TAK1 phosphorylation (45). These findings suggest that disrupting TAK1’s regulation of SnoN can effectively attenuate fibrotic responses.

### Smad independent pathways

The TGF-β pathway is a major driver of fibrosis, promoting the differentiation of fibroblasts into myofibroblasts, which are key effectors in ECM production and tissue stiffening (46–48). TAK1 is activated downstream of TGF-β receptors and works in parallel with the canonical Smad-dependent pathway. For instance, TAK1 activation enhances the phosphorylation of p38 MAPK and JNK, both of which contribute to the transcriptional upregulation of fibrotic genes such as collagen and fibronectin (25, 49–53).

## TAK1 regulation in key SSc cell subtypes driving fibrosis

### Mesangial cells

Mesangial cells play a vital role in maintaining glomerular function and structure, but their dysregulation contributes to kidney fibrosis, a complication of systemic sclerosis. Renal fibrosis in SSc is a significant complication, with scleroderma renal crisis affecting approximately 5% to 20% of patients with diffuse cutaneous SSc and 1% to 2% of those with limited cutaneous SSc (54). Clinically, SRC presents with acute kidney injury and progressive oliguric renal failure (55–57). TAK1 mediates TGF-β-induced upregulation of type I and IV collagens and fibronectin, key ECM components involved in fibrotic progression. Studies have demonstrated that TAK1 activation in mesangial cells is essential for TGF-β-driven fibrotic responses in the kidney (58). Other work has shown that in mesangial cells TGF-β1 induces rapid phosphorylation of the TAK1-MKK3 -p38 MAPK pathways. Notably, blocking the TAK1-MKK3 pathway disrupts the TGF-β1-induced up-regulation of pro-α1(I) collagen, while leaving the induction of fibronectin and PAI-1 unaffected (59–61).

### Fibroblasts

In SSc, fibroblasts undergo phenotypic changes that contribute to disease progression. These alterations include increased proliferation, enhanced collagen synthesis, and decreased collagenase activity, leading to excessive ECM deposition. Additionally, SSc fibroblasts show abnormally shortened primary cilia which is directly linked to the constitutively activated phenotype (62). The fibrotic phenotype of SSc fibroblasts varies with disease duration and severity of skin involvement, as observed in tissue engineering models (63). Additionally, profibrotic fibroblasts may differentiate from normal resident fibroblasts or expand through recruitment of bone marrow-derived progenitors, with multiple factors, signaling pathways and chromatin remodeling involved in the development or persistence of the SSc fibroblast phenotype (64). TGF-β-induced fibronectin expression in fibroblasts is mediated by TAK1 through the MKK4-JNK signaling cascade. Inhibition or deletion of TAK1 in fibroblasts results in a reduced profibrotic response to TGF-β stimulation, highlighting its critical role in driving fibrosis (25). Several studies have linked TAK1 activation with myofibroblast differentiation, a hallmark of SSc pathogenesis, suggesting that TAK1 inhibitors may hold therapeutic potential in mitigating fibroblast-driven fibrosis (21, 25, 65).

### Hepatocytes

Although clinically significant hepatic fibrosis appears to be relatively uncommon in SSc, liver involvement may occur in a subset of patients. In a transient-elastography study of 59 patients with SSc, 3.4% exhibited liver stiffness values consistent with marked fibrosis; importantly, the authors noted that this prevalence was comparable to that expected in the general population (66). Hepatocytes, the principal parenchymal cells of the liver, rely on TAK1 signaling to maintain cellular survival and tissue homeostasis. Hepatocyte-specific deletion of *Tak1* in mice causes spontaneous hepatocyte death, compensatory proliferation, hepatic inflammation, fibrosis, and ultimately hepatocellular carcinogenesis (24). These pathological effects are partially mediated through tumor necrosis factor receptor 1 signaling, indicating that TAK1 protects hepatocytes from inflammatory cell-death pathways (24).

TAK1 can also transmit noncanonical signals downstream of TGF-β and other inflammatory receptors; however, TGF-β has context-dependent effects in hepatocytes and can promote growth arrest or apoptosis rather than uniformly supporting cell survival (67, 68). Accordingly, the available evidence supports TAK1 as an important regulator of hepatocyte survival and inflammatory homeostasis, but it does not yet establish a hepatocyte-specific TAK1 mechanism in SSc-associated liver fibrosis.

### Cardiomyocytes

Cardiac involvement is an important manifestation of SSc, and myocardial inflammation, microvascular injury, and fibrosis can contribute to ventricular dysfunction and heart failure (69). In cardiomyocytes, TGF-β activates both canonical Smad signaling and noncanonical pathways involving TAK1 (70). TAK1 is activated rapidly following TGF-β stimulation and can interact directly with the TGF-β type II receptor, providing a mechanistic link between TGF-β receptor signaling and cardiomyocyte hypertrophic responses (71). In a murine pressure-overload model, cardiomyocyte-specific disruption of TGF-β receptor signaling suppressed myocardial TAK1 activation and protected against maladaptive hypertrophy, ventricular dilation, and dysfunction, supporting a role for cardiomyocyte TGF-β–TAK1 signaling in pathological cardiac remodeling (70).

The effects of TAK1 in the heart are nevertheless context- and cell-type-dependent. Excessive TAK1 activation can promote inflammatory and hypertrophic signaling, and pharmacological suppression of TAK1 has reduced cardiac inflammation, fibrosis, and dysfunction in several experimental models (72). Conversely, cardiomyocyte-specific deletion of TAK1 causes myocyte death, myocardial fibrosis, ventricular remodeling, and heart failure, demonstrating that basal TAK1 activity is also essential for cardiomyocyte survival (73).

## TAK1 and inflammatory cytokines in fibrosis

Inflammatory cytokines, including TNF-α and IL-1β, as well as endogenous ligands of TLR4, are key drivers of chronic inflammation that predispose tissues to fibrosis (**Table 1**). TAK1 is a critical mediator of NF-κβ activation in response to these cytokines, linking inflammation to fibrotic processes. Persistent TAK1 activation maintains a pro-inflammatory environment, which stimulates fibroblast proliferation and ECM production.

**Table 1:** TAK1 agonists.

Activator	Downstream effectors	Impact on fibrosis	Cell types studied	Ref.
TGF-β	p38 MAPK, JNK, NF-κβ	Induces fibrosis by promoting extracellular matrix production and fibroblast activation.	Mesangial cells, fibroblasts	(20)
TNF-α	NF-κβ, JNK, p38 MAPK	Links inflammation to fibrosis by enhancing pro-inflammatory and profibrotic signaling pathways.	Hepatocytes, hepatic stellate cells	(74)
IL-1β	NF-κβ, JNK	Promotes fibrotic remodeling in tissues by upregulating pro-fibrotic gene expression.	Various cell types	(75)
Lipopolysaccharides (LPS)	MyD88, TRIF, NF-κβ, p38 MAPK	Induces fibrosis by promoting inflammation-driven ECM deposition.	Lung fibroblasts, macrophages	(76)

For example, a study by Ma et al. (77) explored the role of TAK1 in renal fibrosis. In a unilateral ureteral obstruction mouse model, TAK1 inhibition reduced the expression of NF-κβ target genes involved in inflammation, such as TNF-α and MCP-1 (77). This reduction in inflammatory signaling correlated with decreased fibroblast activation and ECM deposition, demonstrating that TAK1 bridges inflammatory and fibrotic pathways in kidney disease.

## Pharmacological inhibitors of TAK1

TAK1 has emerged as a critical target for the treatment of inflammatory and immune-related diseases, with multiple inhibitors being developed to probe its therapeutic potential. Covalent inhibitors such as 5Z-7-oxozeaenol (5ZO) and hypothemycin have been widely used in research due to their high potency (78, 79). 5ZO, with an IC_50_ of 9 nM, is the most commonly used covalent TAK1 inhibitor (78). Despite its effectiveness in suppressing inflammation in models of diseases like rheumatoid arthritis, 5ZO suffers from significant off-target effects, inhibiting over 50 kinases (80). Similarly, hypothemycin has an IC_50_ of 100 nM but also shows poor selectivity, targeting kinases such as MEK (79, 81, 82). These off-target liabilities limit the therapeutic applicability of covalent TAK1 inhibitors, despite their utility in preclinical studies (15).

Type I inhibitors, which bind the ATP-binding pocket in the active DFG-in conformation of TAK1, include AZ-TAK1 and ABC-FP. AZ-TAK1 demonstrates an IC50 of 8 nM and has shown efficacy in acute myeloid leukemia models, but it exhibits off-target activity against other kinases like HIPK2 and CDK9 (83). Similarly, ABC-FP binds TAK1 with an IC_50_ of 28 nM but has not undergone extensive selectivity profiling, leaving its broader kinome effects unclear. These inhibitors often face limitations in therapeutic use due to competition with intracellular ATP, which diminishes their efficacy *in vivo* (84). Type II inhibitors, which bind the inactive DFG-out conformation of TAK1, are considered a more selective class. Among them, NG-25 is a dual inhibitor of TAK1 and MAP4K2, with an IC_50_ of 4 nM (85). While its potency is impressive, its dual-target activity raises concerns about off-target effects in therapeutic applications. Another type II inhibitor, PF-05381941, binds TAK1 with an IC_50_ of 156 nM (86). Type II inhibitors can exhibit greater kinase selectivity than type I inhibitors, potentially because they exploit the DFG-out conformation and an adjacent allosteric pocket containing residues that are less conserved across the kinome (87, 88).

The discovery of takinib, a type 1.5 inhibitor, represents a major advancement in TAK1 inhibitor development. Takinib has an IC_50_ of 9.5 nM and exhibits remarkable selectivity, with a 12-fold preference for TAK1 over its next kinase target, IRAK4 (84, 89, 90). Its binding mode combines features of type I and type II inhibitors, offering prolonged target engagement and reduced off-target effects. Despite the initial promise of Takinib, poor solubility limited its pre-clinical and potential clinical development. Following an SAR campaign, a new Takinib analog, HS-276, was developed with improved TAK1 potency, solubility and oral bioavailability (28). Although a co crystal structure confirming the binding motif of HS-276 has yet to be identified, it is predicted to bind as a type 1.5 inhibitor, similar to Takinib, due to the conserved core chemical scaffold of the molecule. At the time of writing, HS-276 was in preclinical development for various autoimmune and inflammatory diseases where promising efficacy in bleomycin mouse model of fibrosis supported its potential clinical use in systemic sclerosis (25–27, 29, 91–93).

## Conclusions

TAK1 has emerged as a central signaling integrator in the pathogenesis of fibrosis and systemic sclerosis, positioned at the convergence of inflammatory, profibrotic, and stress-responsive pathways that drive disease progression. Across multiple cell types implicated in SSc—including fibroblasts, mesangial cells, cardiomyocytes, and immune populations—TAK1 coordinates extracellular signaling inputs from cytokines and growth factors to regulate NF-κβ and MAPK activation, modulate Smad-dependent transcriptional programs, and shape the persistence of myofibroblast activation and ECM deposition. This integrative role places TAK1 not merely downstream of individual stimuli, but at a critical systems-level node governing the transition from adaptive inflammation to maladaptive fibrotic remodeling.

Importantly, the context-dependent effects observed in genetic deletion versus pharmacologic inhibition models underscore the nuanced biology of TAK1. Beyond its catalytic activity, TAK1 functions as a scaffold within multiprotein complexes, and its complete ablation may disrupt homeostatic signaling networks in ways that differ fundamentally from selective kinase inhibition. These findings highlight the need for careful therapeutic modulation rather than wholesale suppression, particularly in a disease such as SSc where immune dysregulation, vascular injury, and fibrosis are tightly intertwined.

Advances in the development of selective TAK1 inhibitors, including next-generation type 1.5 compounds with improved specificity and pharmacologic properties, provide renewed translational momentum. Preclinical data demonstrating attenuation of inflammation and fibrosis support the feasibility of targeting this pathway. Moving forward, critical questions remain regarding cell-type specificity, timing of intervention, and long-term effects on immune competence and tissue repair. Addressing these gaps will be essential for defining the therapeutic window in which TAK1 modulation can interrupt fibrotic progression without compromising physiological signaling.

Collectively, current evidence positions TAK1 as a mechanistic bridge linking inflammation to fibrosis in systemic sclerosis. Continued investigation into its signaling architecture and pharmacologic targeting may yield transformative strategies for altering the trajectory of this devastating disease.
